# The unique problem of glaucoma: Under-diagnosis and over-treatment

**DOI:** 10.4103/0301-4738.73677

**Published:** 2011-01

**Authors:** Barun K Nayak, Quresh B Maskati, Rajul Parikh

**Affiliations:** Department of Ophthalmology, PD Hinduja National Hospital & MRC, Mumbai - 4000 016, India. E-mail: editor@ijo.in; 1 Department of Ophthalmology, Maskati Eye Clinic, Mumbai, India; 2 Department of Ophthalmology, Shreeji Eye Institute and Palak’s Glaucoma Care Centre, Mumbai, India

Glaucoma is a chronic progressive optic neuropathy which is characterized by typical optic disc and retinal nerve fiber layer (RNFL) changes with correlating visual field defects wherein intraocular pressure (IOP) is a major risk factor. It is the second leading cause of blindness.[1] About half the glaucoma patients in a community remain undiagnosed worldwide[2] and in our country this figure is around 90%.[3,4] On the one hand nearly 50-90% of true glaucoma patients remain undiagnosed; on the other hand, nearly half of the “glaucoma patients” using ocular hypotensive medication do not need the medications or are over-treated.[5] With the above figures it is obvious that under-diagnosis and over-treatment are quite common. The issue gets more complex due to the high percentage of non-compliant patients. The effects of under-diagnosis are obvious but the implications of over-treatment are far more deleterious as it increases the cost of treatment, affects the quality of life and subjects patients to the risks of side-effects without much gain. The purpose of this editorial is to highlight the issue of under-diagnosis and over-treatment, so that it can be avoided.

Under-diagnosis of glaucoma is either a result of patients not presenting to their ophthalmologist at all or on time, or ophthalmologists missing the diagnosis. Unfortunately, this issue itself is multifaceted. In many cases, patients do not understand the crucial importance of preventive eye care or are not aware of their own risk for glaucoma. In other cases, some patients do not have access to professional eye care because of insufficient financial resources or no means of transportation.[3,4,6–8]

In the Chennai Glaucoma Survey, nearly 50% of newly diagnosed glaucoma patients had an eye checkup in the previous year, of this less than 20% were diagnosed with glaucoma.[9] In the same survey, authors also reported that 40% of diagnosed open-angle glaucoma patients actually had angle closure.[9] The treating ophthalmologist had missed angle closure because of either the inability to perform the test or not performing a comprehensive ophthalmic examination.

Initiatives to increase public awareness and comprehensive eye examinations by ophthalmologists are the key to reducing or eliminating undiagnosed glaucoma. If all ophthalmologists perform comprehensive eye examinations (that includes basic slit-lamp examination, intraocular pressure (IOP) measurement, pachymetry, gonioscopy and dilated fundus examination), we can definitely minimize under-diagnosis.

The reasons for over-treatment are many. Improper workup and lack of infrastructure in the clinic coupled with fear of blindness due to glaucoma end up in over-treatment in many situations. It is not uncommon to find prescriptions for multiple topical drugs for glaucoma with no disc and field changes with a history of the highest IOP recorded being less than 25 mm of Hg. Many solo practitioners use only tonometer as the basis for treatment. In this scenario the ophthalmologists overestimate the benefit of therapy while underestimating the associated risk of treatment. Introduction of newer molecules and hard selling by pharmaceutical companies compound the problem. Companies are biased in funding and publishing more research involving their products, especially the costly ones.[10–12] Lack of an evidence-based approach adds to the problem. There are no neuroprotective agents approved for commercial use; still many drugs are used erroneously as neuroprotective agents by the physician. At times, a doctor owning a costly laser machine may perform laser procedures when it may not be indicated.

Over-reliance on newer glaucoma diagnostics (such as Heidelberg retinal tomogram, optical coherence tomography, and scanning laser polarimetry) leads to over-diagnosing glaucoma if it is interpreted in isolation without taking into consideration the complete clinical picture.[13] At the slightest hint ophthalmologists either initiate or enhance treatment, but they are reluctant in withdrawing or reducing antiglaucoma medications. Authors (BKN, QBM, RP) spend a lot of time in attempting to withdraw ‘unnecessary’ antiglaucoma medication by performing a ‘reverse therapeutic trial’.

Under-treatment is another issue and is usually encountered in advanced stages of the disease. The advanced glaucoma intervention study (AGIS) clearly suggests that consistently low IOP in lower teens prevents further damage. Although many medications are available in our armamentarium, there is a limitation of IOP coming down below a level with medication alone, hence surgery is the answer. However, surgery is deferred for fear of complications such as macular wipeout in advanced glaucoma. The reported incidence of this complication is only 1-2%. Javitt *et al*., reported that the glaucoma surgery rate among African Americans was 45% lower than the other American population and may be one of the reasons for higher glaucoma blindness in these people.[14] The root cause of under-treatment is complex and may be region-specific or religion-specific.

The problem of under-diagnosis and over-treatment can be tackled by a comprehensive eye examination, creating proper infrastructure, and understanding the published literature in the right perspective. There are many landmark studies published now in glaucoma. Ocular hypertension treatment study (OHTS) provides guidelines for the management of ocular hypertensive patients. The evidence is clear: not all patients with IOP > 21 mm Hg require treatment. Early manifest glaucoma treatment study (EMGT) helps us in deciding treatment in early glaucoma subjects, whereas AGIS guides us in the management of patients with advanced glaucoma. However, to understand these studies in the right perspective, we have to be familiar with certain terminologies such as ‘relative risk reduction’, ‘absolute risk reduction’, ‘number needed to treat’ and ‘number needed to harm’.[15] Clear understanding of this statistical jargon can help us in the appropriate management of glaucoma. I would strongly recommend everyone to attend the ‘Research Methodology Workshop’ conducted by the *Indian Journal of Ophthalmology* at regular intervals.

I wish this editorial will initiate a deep thinking process amongst the ophthalmologists so that under-diagnosis and over-treatment can be minimized. The definition has changed but we are still stuck with 21 mm Hg as the magic number. The moment IOP crosses this “magic number”, the ocular hypotensive medications are started even if IOP is checked on non-contact air puff tonometers. If the IOP is normal, then the rest of the examination is not performed. A careful comprehensive examination may pick up a glaucomatous disc which can exist with “a statistically normal” IOP.

A statement often used by a learned professor “we smell cataract and forget about everything else and when we can’t even smell cataract, we start antiglaucoma medications”, needs a proper retrospection in light of this editorial.
